# Short-term effects of a rights-based sexuality education curriculum for high-school students: a cluster-randomized trial

**DOI:** 10.1186/s12889-015-1625-5

**Published:** 2015-03-26

**Authors:** Norman A Constantine, Petra Jerman, Nancy F Berglas, Francisca Angulo-Olaiz, Chih-Ping Chou, Louise A Rohrbach

**Affiliations:** Center for Research on Adolescent Health and Development, Public Health Institute, 555 12th Street, 10th Floor, 94607 Oakland, CA USA; Division of Community Health and Human Development, School of Public Health, University of California, Berkeley, 50 University Hall, 94720 Berkeley, CA USA; Institute for Health Promotion and Disease Prevention Research, Keck School of Medicine, University of Southern California, 2001 N. Soto Street, 90032 Los Angeles, CA USA

**Keywords:** Adolescent, Sexuality education, Comprehensive sexuality education, Sexual behavior, Sexual rights, Gender norms, Intervention, Evaluation

## Abstract

**Background:**

An emerging model for sexuality education is the rights-based approach, which unifies discussions of sexuality, gender norms, and sexual rights to promote the healthy sexual development of adolescents. A rigorous evaluation of a rights-based intervention for a broad population of adolescents in the U.S. has not previously been published. This paper evaluates the immediate effects of the Sexuality Education Initiative (SEI) on hypothesized psychosocial determinants of sexual behavior.

**Methods:**

A cluster-randomized trial was conducted with ninth-grade students at 10 high schools in Los Angeles. Classrooms at each school were randomized to receive either a rights-based curriculum or basic sex education (control) curriculum. Surveys were completed by 1,750 students (*N* = 934 intervention, *N* = 816 control) at pretest and immediate posttest. Multilevel regression models examined the short-term effects of the intervention on nine psychosocial outcomes, which were hypothesized to be mediators of students’ sexual behaviors.

**Results:**

Compared with students who received the control curriculum, students receiving the rights-based curriculum demonstrated significantly greater knowledge about sexual health and sexual health services, more positive attitudes about sexual relationship rights, greater communication about sex and relationships with parents, and greater self-efficacy to manage risky situations at immediate posttest. There were no significant differences between the two groups for two outcomes, communication with sexual partners and intentions to use condoms.

**Conclusions:**

Participation in the rights-based classroom curriculum resulted in positive, statistically significant effects on seven of nine psychosocial outcomes, relative to a basic sex education curriculum. Longer-term effects on students’ sexual behaviors will be tested in subsequent analyses.

**Trial registration:**

ClinicalTrials.gov NCT02009046 [http://www.advocatesforyouth.org/the-3rs].

## Background

Despite sharp declines since its peak in the 1990s [1], the teen birth rate in the United States remains high relative to many other developed countries [2,3], and marked disparities persist by racial and ethnic group [1,4]. Moreover, rates of many sexually transmitted infections (STIs) are particularly high among adolescent and young adult populations, potentially causing long-term health problems and contributing to high health care costs for screening and treatment [5,6]. Recent decades have seen a range of efforts aimed at reducing rates of teen births and STIs, and more than 95% of U.S. adolescents now report having received some formal sexuality education in a school, church or community setting by age 18 [7]. The content and format of formal sexuality education interventions have varied greatly over time, with the current emphasis centered on school-based programs that provide instruction on abstinence from sexual activity plus the use of contraception and condoms for those who are sexually active [8-12].

Among adolescent sexual health scholars and advocates in the U.S. and globally, discussions around sexuality education are increasingly being framed more broadly—embracing comprehensive approaches based on frameworks of positive sexual health promotion and youth development and going beyond the typically more limited focus on pregnancy and disease prevention [12-15]. One emerging model is the rights-based approach, which seeks to unify issues of sexuality, human rights, and gender to promote healthy sexual development [16]. More specifically, the rights-based approach is guided by a recognition of adolescents’ fundamental rights to sexual health information and services, self-determination, and non-discrimination, which also are core to frameworks of reproductive rights [17,18] and reproductive justice [19]. It expands the goals of sexuality education beyond disease and pregnancy prevention to include positive sexuality, empowerment, and even civic engagement, and incorporates curriculum content related to the larger contextual issues that affect adolescents’ sexual lives, including gender and cultural norms, relationship power, and sexual orientation.

Although a rights-based framework has been referenced in a number of international standards and guidelines [20-22] and some U.S. advocacy efforts [23], the development of programs that operationalize these concepts is still at a nascent stage, particularly in the United States. One resource has been the Population Council’s 2009 *It’s All One* kit of curriculum guidelines and activities [24], and documentation of its use in a variety of settings is underway [25]. There have been descriptive field reports of positive experiences from rights-based programs around the world [25-27]. In addition, research studies of conceptually related interventions (such as those that have incorporated discussions of gender norms) have been cited as evidence supporting the approach [28-33]. These more rigorous studies, which include randomized controlled trials and a meta-analysis, have found positive effects on sexual health outcomes, but may be limited in their applicability to adolescents in U.S. schools due to differences in country contexts, intervention settings, and/or target populations. For example, most have focused on young adult populations, and those studies that have centered on adolescents typically have been limited to sexually active females presenting in clinic settings. To our knowledge, no rigorous evaluation has been published of a rights-based sexuality education intervention for adolescents in the United States.

### The present study

We conducted a cluster-randomized trial of a multicomponent, rights-based sexuality education intervention delivered to students enrolled in high schools in low-income, urban communities of Los Angeles. The primary aim of the trial was to determine the effectiveness of a new rights-based classroom curriculum. The secondary aim was to evaluate the impact of the multicomponent intervention, which included the curriculum as well as parent education workshops and materials, a peer advocate program, and access to sexual health services. In this article we examine the effects of the classroom curriculum, compared to a control curriculum, on short-term psychosocial outcomes including sexual health knowledge, attitudes and communication. These short-term psychosocial outcomes are expected to be mediators of longer-term effects on students’ sexual behaviors, which will be tested in future analyses. We further examine differences in intervention effects by student gender and baseline sexual experience, two factors often considered as moderators of impact [8,9]. The secondary aim of the full study regarding the impact of the full multicomponent intervention will be addressed in a subsequent report based on the one-year follow-up data.

### The intervention

The Sexuality Education Initiative (SEI) was developed by Planned Parenthood Los Angeles (PPLA) with the goal of improving the sexual and reproductive health of low-income, primarily Hispanic and African American youth in Los Angeles high schools. The SEI was designed to reach these goals by reducing students’ risk of pregnancy and STIs, as well as improving students’ ability to manage their sexuality respectfully. It employed a rights-based framework that focuses on human rights, gender equality, access to health care services, and critical thinking, and emphasized the relationship between broader social and cultural factors and individuals’ sexual decisions. PPLA designed the SEI content and format on the basis of best practices outlined by international and U.S. organizations, as well as findings from formative research and pilot testing with the target community. A detailed overview of the influences, decisions, and processes related to program development is presented elsewhere [27].

The SEI was comprised of four components, one implemented at the classroom level and three implemented at the school level. At the classroom level, a 12-session rights-based curriculum for ninth-grade students addressed issues of gender roles and power dynamics in relationships and in media messages, and emphasized sexual rights, in addition to providing more commonly available content on sexual and reproductive anatomy, pregnancy, STIs/HIV, and contraception (see Table 1). The curriculum included the use of interactive techniques, such as small group exercises, classroom discussion, and critical thinking activities.

**Table 1:** **Overview of Sexuality Education Initiative (SEI) 12-session classroom curriculum topics**

**Lesson**	**Topics**
1. Introduction	Overview of program goals; introduction to gender stereotypes; availability of clinical sexual health services
2. Social and media messages	Images of femininity, masculinity, sex, and sexuality in popular media; impact of media on body image and gender-based violence
3. Gender and identity	Gender roles and stereotypes; how gender roles change over time; how strict gender roles can affect relationships
4. Relationships	Rights and responsibilities in sexual relationships; signs of healthy and unhealthy relationships
5. Sexuality	Defining sex and sexuality; sexuality as healthy and normal part of life; choosing abstinence and reasons to delay sex
6. Sexual and reproductive anatomy	Male and female anatomy; understanding how bodies work and normal differences in bodies’ shapes, sizes, and colors
7. Pregnancy	Biology of conception and pregnancy; pregnancy options; reasons for becoming or not becoming a parent
8. STIs and safer sex	How STIs are transmitted; differences between curable and treatable STIs; practicing safer sex; condom demonstration
9. HIV/AIDS	HIV/AIDS transmission, prevention and testing; continuum of risk for sexual behaviors
10. Contraception	Common methods of pregnancy prevention; effectiveness and safety of methods; how gender norms may affect decisions about sex; partner communication
11. Sexual choice and coercion	Unwanted vs. wanted sexual activity; consent; right to say ‘no’ and responsibility to ask; knowing one’s sexual limits; partner communication
12. Decision-making	Making healthy decisions about sex and relationships; how gender stereotypes may affect decision-making; identifying future goals

The three school-level SEI components included parent education workshops and materials, a peer advocate program, and access to sexual health services. Educational workshops for parents addressed adolescent sexuality, teen pregnancy, STIs, healthy relationships, values, and parent-teen communication. An after-school peer advocacy program offered intensive training and leadership skill-building for selected students, who in turn planned sexual health awareness events and publicized available health services on campus to their classmates. Sexual health services were made available through a “clinic without walls” model, in which PPLA staff visited the regularly throughout the year and provided confidential and youth-friendly services (e.g., pregnancy and STI testing, counseling, prescriptions for contraceptives, and referrals). PPLA staff also distributed packets of free condoms to students and provided training to school staff to become condom distributors on their school campus.

The conceptual framework (theory of change) guiding the SEI design is shown in Table 2. At the broadest level, the SEI was designed to affect psychosocial outcomes—students’ sex- and sexuality-related knowledge, attitudes, communication, self-efficacy, and behavioral intentions—in the short-term and long-term, and sexual behaviors in the long-term. In the present paper, we focus on short-term outcomes, which are expected to be mediators of longer-term effects on sexual behaviors.

**Table 2:** **Sexuality Education Initiative (SEI) conceptual framework (theory of change)**

**Components**	**Short-term outcomes (Mediators)**	**Long-term outcomes**	**Goals**
1. Classroom curriculum	1. Increase understanding that men and women have equal rights regarding sexual relationships and sexual and reproductive health	1. Reduce pregnancy risk, the percentage of youth who report engaging in vaginal sexual intercourse but not using an effective method of contraception during the previous three months	1. Improve the sexual and reproductive health of youth attending Los Angeles high schools
2. Parent education workshops	2. Increase communication about relationships, rights, and sexuality with parents, guardians, or other trusted adults	2. Reduce STI risk, the percentage of youth who report engaging in oral or vaginal sexual intercourse but not using a condom during the previous three months	2. Improve the ability of youth attending Los Angeles high schools to manage their sexuality respectfully
3. Peer advocate program	3. Increase communication about relationships, rights, and sexuality with partners	3. Reduce number of sexual partners	
4. Access to sexual health services	4. Increase knowledge about sex, sexuality, and sexual risk protection	4. Increase use of sexual and reproductive health services	
	5. Increase self-efficacy to assert sexual limits and to manage risky situations		
	6. Increase intentions to protect self from sexual risk		
	7. Increase access to accurate information about sexuality and sexual health		
	8. Increase access to and awareness of sexual and reproductive health services		

## Methods

### Study design

The study was conducted with ninth-grade students enrolled at 10 high schools in low-income, primarily Hispanic communities of Los Angeles. Ten charter schools affiliated with a local school district were selected and recruited to reflect the overall profile of students in the local communities while offering a smaller, contained environment in a charter school setting to conduct intervention research. School administrators agreed to the randomization of intervention components, delivery of the intervention, administration of the survey instrument, and restriction of similar programs on site for the duration of the study period. Following a one-year pilot test (2010-11) the intervention was delivered across two school-year cohorts. In Year 1 (2011-12), eight high schools participated in the study. Following the first year, one matched pair of schools dropped out of the study due to school administration changes and a new matched pair of schools with similar student characteristics was added for Year 2 (2012-13). Thus, ten schools participated in intervention delivery over the two-year period.

Schools were randomized within five pairs of schools matched on percentages of Hispanic and African-American students, and students receiving free lunches. Within each matched pair, one school was randomly assigned to receive all three school-wide SEI components (parent education, peer advocacy, and sexual health services) and the other to receive only the sexual health services school-wide component. Within each school, ninth-grade classrooms were randomized to receive either the 12-session SEI curriculum described above, or a 3-session control curriculum covering basic sexual health topics, including anatomy and prevention of unintended pregnancy and STIs. The control curriculum had been widely implemented by PPLA in previous years and reflected the “standard of care” for sexuality education in local high schools. Both SEI and control curriculum sessions were developed to fit into 50-minute class periods. The SEI sessions were administered across an average span of 53 days, and the control sessions across an average span of 9 days. The SEI curriculum was taught by PPLA staff who participated in a 2-day training on the rights-based curriculum; the control curriculum was taught by PPLA volunteer educators who participated in a 1-day training on the basic curriculum. Regular classroom teachers were present at all sessions.

### Ethical clearance

The study was conducted in compliance with the institutional review boards of the University of Southern California and the Public Health Institute. Positive parental informed consent and student assent were required prior to students’ participation in the evaluation.

### Data collection procedures

Students whose parents provided consent and who assented to participate were administered a written survey at baseline (pretest), immediately following curriculum implementation (posttest), and at one-year following curriculum completion (follow-up). Surveys were administered and collected by study staff at single classroom sessions during regular school hours. Posttest surveys were administered within two weeks of the final session for both curricula. Students absent on testing days were left absentee packets, and asked by school staff to complete the survey in a private area of the classroom, place it a sealed envelope, and return it to the teacher for transmittal to evaluation staff.

To examine fidelity of program implementation, trained members of the research team conducted formal observations of intervention and control curriculum sessions. Across the two-year study period, 220 intervention sessions and 43 control sessions were observed (equivalent to 50% of all sessions in Year 1, and 25% of all sessions in Year 2), with coverage across all schools and sessions. Additionally, student attendance was tracked by PPLA or school staff at all sessions.

### Participants

**Figure 1:**
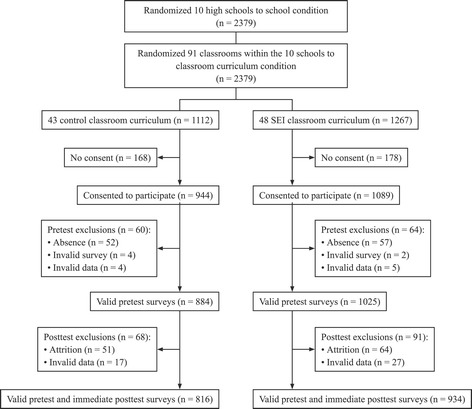
**Participant flow from randomization to final analysis sample.**

Figure 1 displays the flow of participants in the present study, which focused on baseline pretest and immediate posttest surveys. Of 2,379 eligible students in the 91 classrooms at the 10 participating schools, 2,033 (85.5%) consented to participate. Consent rates were similar for intervention (85.9%) and control (84.9%) students. At pretest, 5.7% of cases (5.9% in control group and 5.4% in intervention group) were excluded due to the student’s absence or invalid survey (e.g., blank or mostly blank). In addition, 0.5% of the remaining cases (0.5% in control group and 0.5% in intervention group) were excluded due to invalid data (e.g., multiple inconsistencies across responses). At the immediate posttest, 6.0% of cases were lost due to attrition (5.8% in control curriculum group and 6.2% in SEI curriculum group). In addition, at the immediate posttest, 2.5% of the remaining cases were excluded due to invalid data (2.0% in control group and 2.8% in intervention group). The final pretest–immediate posttest merged dataset included a total of 1,750 students, with 934 students in 48 SEI curriculum classrooms and 816 students in 43 control classrooms.

### Measures

#### Survey development

The student survey was developed following a multistep process. Items were identified in the research literature and reviewed for appropriateness for the target population and intervention objectives. New measurement scales of rights- and gender-based constructs were developed based on formative research with youth and parents [34] and a comprehensive review of the developing classroom curriculum. One-on-one cognitive interviews were conducted with youth to assess comprehension of the items and evaluate the quality of responses, after which the survey was revised accordingly. A pilot version of the survey was administered to more than 700 ninth-grade students at six high schools in the target communities in the year prior to program implementation. Psychometric analyses of reliability and validity were conducted based on these responses, leading to further revisions to the survey items and scales. Additionally, the pilot testing revealed that some students could not complete the entire survey during the available 50-minute class period. To address this challenge we reduced the survey length and student burden by creating two parallel shorter survey forms, with each form randomly administered to half the students in the sample. As a result, a small number of items and scales (described below) appeared on only one of the two survey forms.

#### Psychosocial outcomes

The following survey measures were used to assess the nine short-term psychosocial outcomes (mediators) that were established in the framework guiding the SEI design.

##### Attitudes about rights in sexual relationships

Two 17-item scales assessed attitudes about one’s sexual relationship rights with a steady partner, or with a casual partner (e.g., “With [a steady partner (like a boyfriend or a girlfriend)/someone I just met], a person always has the right to stop having sex with their partner at any time.”). For each scale, the items were averaged, and the coefficient alphas were .89 and .90, respectively. Each set of items was included on one version of the survey and, therefore, was answered by a random half of the sample. For each item, students indicated their agreement on a 4-point Likert-type scale (strongly disagree to strongly agree). A higher mean score indicated more-positive attitudes about one’s rights in sexual relationships.

##### Communication about relationships, rights, and sexuality with partners

Sexual communication with one’s current or most recent steady partner was measured by a 9-item scale (e.g., “Did you ever talk with your current or recent steady partner about whether or not to have sex?”). The items were summed, creating a total score that ranged from 0 to 9, with a higher score indicting a greater number of topics discussed.

##### Communication about relationships, rights, and sexuality with parents/guardians

Sexual communication with one’s parent or guardian was measured by a 15-item scale (e.g., “Have you ever talked with your parent or guardian about oral sex?”). The items were summed, creating a total score that ranged from 0 to 15, with a higher score indicating a greater number of topics discussed. The scale items were included on only one version of the survey and, therefore, were answered by a random half of the sample.

##### Knowledge about sex, sexual health, and sexual risk protection

Sexual health knowledge was assessed using 17 items (e.g., “If a condom is used correctly, it helps protect against HIV.”). For each item, students indicated whether they believed the statement to be true or not true. The items were summed, creating a total score that ranged from 0 to 17, with a higher score indicting a greater number of correct items.

##### Self-efficacy to assert sexual limits and manage risky situations

Students’ protection self-efficacy was measured by a 6-item scale (e.g., “I know how to say ‘no’ to sex.”). The items were averaged, and the coefficient alpha was .78. For each item, students indicated their agreement on a 4-point Likert-type scale. A higher mean score indicated higher self-efficacy.

##### Intentions to protect oneself from sexual risk through condom use

Students’ intentions to use condoms with a sexual partner were measured by a 3-item scale (e.g., “If I had sex with someone, we would use a condom.”). The items were averaged, and the coefficient alpha was .84. For each item, students indicated their agreement on a 4-point Likert-type scale. A higher mean score indicated a greater level of condom-use intentions.

##### Access to accurate information about sexuality and sexual health

A single item assessed whether students knew of a person or place where they could access good information about sexuality or sexual health. For these analyses, responses were dichotomized as “yes” or “no/not sure”.

##### Awareness of sexual and reproductive health services

A single item assessed whether students knew where they could access sexual health services (defined as birth control, pregnancy tests, and STI tests). For these analyses, responses were dichotomized as “yes” or “no/not sure”.

### Student characteristics

Additional survey items assessed student demographic and behavioral characteristics at baseline. *Gender* was measured dichotomously. Household crowding was used as a proxy for *socioeconomic status*, and was measured as the ratio of the number of rooms in the student’s home by the number of people living in the home [35]. *Acculturation* was measured using an adapted version of a brief scale developed by Marin and colleagues [36] and validated with Hispanic adolescents [37]; we calculated a mean score across four items that assessed the extent to which the student relied on English or another language when reading, speaking with friends, watching movies or television, and speaking at home [36,37]. Responses were measured on a 5-point scale ranging from “only English” (1) to “only another language (besides English)” (5); for data analyses, the responses were reverse coded. Students were also asked whether they were *born in the United States. Sexual experience* was measured dichotomously; students were asked if they ever had sex, with sex defined as vaginal or anal sex. Students’ *age* and *race/ethnicity* were collected, but not used in these analyses due to limited variation in responses.

### Fidelity of implementation

This paper presents three measures of fidelity of implementation: 1) student attendance (tracked by project staff), 2) delivery of all sections and activities in a session according to the lesson plan (based on observations), and 3) delivery of session content according to the lesson plan (based on observations). Attendance data were summarized as the mean percentage of students in attendance at each session in a given classroom. Completion of session sections and activities by the educator was assessed by trained members of the research team during formal classroom observations and summarized as the percentage of sections and activities delivered. Delivery of session content according to the lesson plan was assessed during classroom observations and measured on a scale of 1 (“not at all closely”) to 5 (“very closely”).

### Analyses

Attrition analyses were conducted to determine whether the attrition rate was comparable across curriculum intervention groups and to examine whether students who were lost to follow-up differed on key characteristics from those who were retained. Students’ demographic and baseline behavioral characteristics were compared between the two curriculum intervention groups.

Comparisons of pretest–posttest changes in outcome measures by curriculum intervention group were conducted using multilevel linear and logistic regression models (using SAS 9.2 PROC MIXED and GLIMMIX procedures) to account for the interdependence among clustered student observations due to classroom and school affiliations [38-40]. Statistical significance levels of p < .05 were employed across all analyses. The short-term outcomes tested were posttest scores on the nine short-term psychosocial measures described above. To account for interdependence at the school level (10 schools), a school identification variable was entered as a fixed effect. To account for interdependence at the classroom level (91 classrooms), a classroom identification variable was entered as a random effect. Classroom curriculum intervention group (control vs. SEI) was included as a fixed effect. Student level covariates included gender, household SES, acculturation, baseline sexual experience, and the pretest measure of the tested outcome. The two continuous covariates—household SES and acculturation—were group-mean centered (i.e., centered on the variable’s classroom mean).

We examined two cross-level interactions for all nine short-term outcomes to examine differential intervention effect by gender and sexual experience. The first interaction was between curriculum intervention group and student gender (individual-level), and the second one between curriculum intervention group and student baseline sexual experience (individual-level). Furthermore, classroom mean gender and classroom mean baseline sexual experience were included in the models as classroom-level covariates to reflect the environmental influence of the classroom (contextual effects); both were centered by subtracting a constant (i.e., the mean across all the classroom means) from each classroom’s mean. As part of examining these contextual effects, we also added two classroom-level interactions to the models. The first interaction was between curriculum intervention group and classroom mean gender, and the second one between curriculum intervention group and classroom mean sexual experience. As variables were added to the regression models, we tested model fit by examining statistically significant differences in model deviance. When a subsequent model did not show an improved fit over a previous model, the previous model was chosen as the final model for interpreting results.

For the seven continuous outcomes, effect sizes were represented by adjusted standardized mean differences, which were calculated by dividing the regression estimate by the within-classroom standard deviation for the final model. The within-classroom standard deviation was used rather than the between-classroom standard deviation because the latter was either zero or near zero in the final models. For the two dichotomous outcomes, effect sizes were represented by odds ratios.

## Results

### Attrition

Comparisons between students lost to attrition and students who were retained were made on seven key background characteristics assessed at pretest (see Table 3). The comparisons revealed that students who were lost to attrition were more likely to be older, non-Hispanic, from a higher household SES, and more likely to have ever had sex at pretest than were students who were retained (p < .05). There were no statistically significant differences by gender or by being born in the U.S. The difference in rate of attrition from pretest to immediate posttest for the control curriculum group (5.8%) relative to the SEI curriculum group (6.2%) was not statistically significant.

**Table 3:** **Comparison of students retained for analysis with students lost to attrition at posttest**

**Variable**	**Retained students**	**Lost to attrition**	**p value**
**Overall (%)**	(N = 1794)	(N = 115)	
Control	94.2	5.8	
SEI	93.8	6.2	0.664
**Gender (%)**	(N = 1786)	(N = 108)	
Male	49.5	47.2	
Female	50.5	52.8	0.646
**Age (12-18 years)**	(N = 1745)	(N = 108)	
Mean (SD)	14.21 (0.60)	14.38 (0.77)	0.026
**Hispanic (%)**	(N = 1690)	(N = 101)	
No	9.2	15.8	
Yes	90.8	84.2	0.027
**Born in the United States (%)**	(N = 1696)	(N = 104)	
No	14.2	9.6	
Yes	85.8	90.4	0.189
**Household SES (0-3)**	(N = 1710)	(N = 104)	
Mean (SD)	0.57 (0.28)	0.63 (0.27)	0.043
**Student acculturation (1-5)**	(N = 1726)	(N = 105)	
Mean (SD)	3.81 (0.71)	3.95 (0.76)	0.051
**Ever had sexǂ (%)**	(N = 1770)	(N = 112)	
No	79.2	53.6	
Yes	20.8	46.4	0.000

ǂDefined as vaginal or anal sex. *Notes:* Difference between groups was tested by chi-square for categorical variables and *t* test for continuous variables, at p < .05. The analyses were not adjusted for interdependence due to classroom and school affiliations.

### Baseline characteristics

Baseline characteristics of students by curriculum intervention group are presented in Table 4. Overall, the SEI curriculum group and the control curriculum group were similar across baseline characteristics, with no statistically significant differences observed. Fifty-one percent of the students in the total sample were female, and the mean student age was 14.20 years. In addition, 85.9% of students identified as Hispanic, and 81.1% were born in the United States. Students in the total sample had a mean household SES of 0.57 on a scale of 0 to 3, and a mean acculturation score of 3.81 on a scale of 1 to 5. Nearly one-fifth (19.0%) reported having had vaginal or anal sex in their lifetime.

**Table 4:** **Baseline characteristics of students by curriculum intervention group**

**Characteristic**	**Total (N = 1750)**	**Control (N = 816)**	**SEI (N = 934)**
**Gender (%)**			
Male	49.1	48.8	49.4
Female	50.6	50.7	50.5
Missing	0.3	0.5	0.1
**Age (12-18 years), mean (SD)**	14.20 (0.60)	14.19 (0.58)	14.22 (0.62)
**Hispanic (%)**			
No	8.6	8.5	8.7
Yes	85.9	85.0	86.7
Missing	5.5	6.5	95.4
**Born in the United States (%)**			
No	13.5	13.2	13.7
Yes	81.1	80.6	81.6
Missing	5.4	6.1	4.7
**Household SES (0-3), mean (SD)**	0.57 (0.27)	0.57 (0.26)	0.57 (0.27)
**Student acculturation (1-5), mean (SD)**	3.81 (0.70)	3.81 (0.70)	3.81 (0.71)
**Ever had sexǂ (%)**			
No	79.6	80.1	79.1
Yes	19.0	18.5	19.5
Missing	1.4	1.3	1.4

ǂDefined as vaginal or anal sex. Notes: Percentages might not add up to 100% due to rounding. Differences between control and intervention classrooms were tested by chi-square for categorical variables and *t* test for continuous variables, at p < .05. The analyses were not adjusted for interdependence due to classroom and school affiliations.

### Overall outcomes

Students’ unadjusted pretest and immediate posttest outcome measure scores in both curriculum intervention groups are shown in Table 5. The adjusted pretest–posttest score differences between the two groups were examined in multilevel linear and logistic regression models. Table 5 shows the results of the final regression model for each outcome.

**Table 5:** **Unadjusted and adjusted outcome measures by curriculum intervention group for nine short-term psychosocial outcomes**

**Outcome measure**	**Control**		**SEI**		**Multilevel regression estimates and effect sizes**		**ICC**
	**Pretest**	**Posttest**	**Pretest**	**Posttest**			
**Continuous measures**					**Estimate (CI)**	**Adjusted standardized mean difference (CI)**	
Rights with steady partner (1–4), mean (SD), N = 783	3.25 (0.41)	3.29 (0.51)	3.21 (0.41)	3.38 (0.50)	0.12 (0.06–0.18)**	0.29 (0.15–0.44)	0.000
Rights with casual partner (1–4), mean (SD), N = 753	3.11 (0.49)	3.14 (0.57)	3.13 (0.46)	3.30 (0.52)	0.19 (0.11–0.27)**	0.42 (0.25–0.59)	0.031
Communication with partners (0–9), mean (SD) N = 1134	2.81 (2.54)	3.24 (2.92)	3.16 (2.75)	3.62 (3.00)	0.14 (-0.13–0.40)	0.06 (-0.06–0.18)	0.000
Communication with parents (0–15), mean (SD), N = 1624	5.68 (4.54)	6.01 (4.99)	5.96 (4.75)	6.70 (5.25)	0.51 (0.10–0.92)*	0.13 (0.03–0.23)	0.000
Sexual health knowledge (1–17), mean (SD), N = 1675	10.90 (2.29)	12.63 (2.16)	10.67 (2.31)	13.40 (2.17)	0.88 (0.65–1.12)**	0.44 (0.33–0.56)	0.031
Self-efficacy to assert oneself (1–4), mean (SD), N = 1545	2.91 (0.56)	3.16 (0.50)	2.93 (0.58)	3.33 (0.52)	0.17 (0.12–0.22)**	0.37 (0.26–0.47)	0.000
Intentions to protect oneself (1–4), mean (SD), N = 1584	3.42 (0.63)	3.43 (0.65)	3.44 (0.60)	3.46 (0.65)	0.03 (-0.03–0.09)	0.05 (-0.05–0.15)	0.008
**Dichotomous measures**					**Logit estimate (CI)**	**Odds ratio (CI)**	
Access to sexual health information (yes), %, N = 1685	44.1%	73.2%	46.1%	86.7%	0.96 (0.68–1.24)**	2.61 (1.97–3.47)	0.012
Awareness of sexual health services (yes), %, N = 1693	49.5%	75.1%	49.6%	87.0%	0.91 (0.62–1.20)**	2.48 (1.85–3.31)	0.009

*p < .05. **p < .001. ICC = intraclass correlation coefficient. CI = confidence interval. Notes: Final models were adjusted for student gender, classroom mean gender, student sexual experience, classroom mean sexual experience, and pretest score. The adjusted standardized mean difference represents the regression estimate divided by the within-classroom standard deviation from the final model for each outcome; the confidence interval for the adjusted standardized mean difference represents the regression estimate’s confidence interval divided by the within-classroom standard deviation from the final model for each outcome.

Results indicated that students in the SEI curriculum group showed larger increases in scores from pretest to posttest than students in the control curriculum group, and that this SEI curriculum group effect was statistically significant for seven of the nine outcome measures. The largest curriculum intervention group effects were found for the scales assessing sexual health knowledge (adjusted standardized mean difference = 0.44), attitudes about rights in sexual relationships with a casual partner (0.42), and self-efficacy to assert sexual limits and manage risky situations (0.37). For the dichotomous outcomes, the odds of students in the SEI curriculum group having access to information about sexuality and sexual health and being aware of sexual and reproductive health services were 2.61 and 2.48 times larger, respectively, than the odds of students in the control group.

### Effects by gender and baseline sexual experience

In examining interactions of curriculum intervention group by student gender and baseline sexual experience for each outcome, we found no statistically significant differential effects (see Table 6). Although student gender had a statistically significant main effect on most of the outcome measures (results not shown), this did not translate into differential effects by curriculum intervention group.

**Table 6:** **Multilevel regression estimates (confidence intervals) for interactions between curriculum intervention group and gender and baseline sexual experience**

**Outcome measure**	**Interaction between curriculum intervention group and**	
	**Student gender**	**Student baseline sexual experience**
Rights with steady partner (N = 783)	-0.02 (-0.14–0.09)	0.04 (-0.11–0.19)
Rights with casual partner (N = 753)	-0.02 (-0.16–0.11)	-0.12 (-0.29–0.06)
Communication with partners (N = 1134)	0.05 (-0.48–0.58)	0.10 (-0.53–0.72)
Communication with parents (N = 1624)	-0.60 (-1.41–0.22)	0.45 (-0.60–1.50)
Sexual health knowledge (N = 1675)	-0.09 (-0.48–0.31)	0.21 (-0.30–0.73)
Self-efficacy to assert oneself (N = 1545)	0.09 (-0.00–0.19)	0.05 (-0.08–0.17)
Intentions to protect oneself (N = 1584)	0.02 (-0.10–0.13)	-0.01 (-0.16–0.15)
Access to sexual health information (yes; N = 1685)	0.31 (-0.24–0.86)	0.23 (-0.50–0.97)
Awareness of sexual health services (yes; N = 1693)	-0.05 (-0.59–0.50)	0.24 (-0.51–0.98)

Notes: None of the interactions was statistically significant. Models were adjusted for curriculum intervention group, student gender, classroom mean gender, student sexual experience, classroom mean sexual experience, and pretest score.

To examine the contextual influence of the classroom, we also included in the models classroom-level measures of these two covariates—classroom mean gender and classroom mean sexual experience—as well as their interactions with curriculum intervention group. We found no statistically significant contextual effect of classroom mean gender for any of the outcomes and no statistically significant interaction effect with curriculum intervention group. We also found no statistically significant contextual effect of classroom mean sexual experience for any of the outcomes, and only one statistically significant interaction effect with the curriculum intervention group. For the final model examining attitudes about rights in sexual relationships with a casual partner, the results indicated a non-significant main contextual effect of classroom mean sexual experience (results not shown). But, the statistically significant interaction (p < .05) with curriculum intervention group showed that the influence of sexual experience was greatly reduced in the SEI curriculum group, relative to the control curriculum group (results not shown).

### Fidelity of implementation

Average student attendance across classrooms was 90.4% for the intervention curriculum and 90.3% for the control curriculum. Most lessons and activities were delivered in their entirety for both the intervention (97.7%) and control (96.0%) classrooms. Mean scores for educator delivery of content according to the lesson plan were 4.52 and 4.39 (on a scale from 1 to 5) for the intervention and control classrooms, respectively.

## Discussion

The results indicate a largely consistent pattern of short-term intervention effects of a new rights-based sexuality education curriculum on ninth-grade students’ knowledge, attitudes, communication, and self-efficacy about sex and sexuality. Students’ scores increased from pretest to posttest for all nine of the short-term outcome measures outlined in the conceptual framework guiding the SEI design. For seven of these outcomes, students who received the SEI’s rights-based classroom curriculum showed statistically significantly greater improvements than did students who received a basic sex education (control) curriculum.

The magnitude of these effects offers additional support to these results. The effect size, as measured by the adjusted standardized mean difference, for four of the five statistically significant continuous outcomes exceeded 0.20, the level considered of policy relevance in educational research [41,42]. In addition, findings for the two dichotomous measures indicated that the odds of SEI curriculum students reporting access to sexual health information and awareness of sexual health services were more than twice as large as for the control curriculum students.

The absence of intervention effects for two of the short-term outcomes might be explained in part by issues of measurement, and in part by the content of each curriculum. Although the control curriculum did not address partner communication as explicitly as the SEI curriculum, it is possible that even basic classroom discussions of reproductive anatomy, pregnancy, contraceptive methods, and STI prevention might raise awareness of the importance of communicating with one’s partner about sexual decision-making. More sensitive measures of frequency and quality of partner communication might lead to the detection of posttest differences between curriculum groups, if they exist. The null intervention effect on students’ intentions to use condoms might be indicative of a ceiling effect in the survey measure. Scores were high for both the intervention and control groups at pretest (3.44 and 3.42, respectively, on a scale of 1 to 4), offering little opportunity for increase due to curriculum participation. In addition, our measure of condom use intentions was comprised of three survey items. The inclusion of more items might broaden the distribution of scores. For both cases of null intervention effects, improvement of survey measures in future research might clarify whether the absence of a statistically significant effect was due to limitations in measurement or the need for greater differentiation between the SEI and a control curriculum.

Notably, the effects of the SEI intervention on short-term outcomes were consistent regardless of student gender or baseline sexual experience, two characteristics often considered as potential moderators of impact in sexuality education interventions. We found no evidence of differential intervention effects in this study. The finding that the SEI had similar short-term outcome effects on both male and female students is important, as this is one of the few rights-based sexual health interventions to address both male and female youth with a common curriculum. The absence of differential intervention effects by gender may reflect, at least in part, the overall intervention approach, which focuses on engaging all students together in discussions of gender, rights, and sexuality. It is also consistent with meta-analyses of adolescent pregnancy prevention interventions that have found no overall differences in impact on behavioral outcomes by gender for pooled estimates [43-45]. Similarly, the lack of differential effect by baseline sexual experience may reflect the deliberate design of the intervention for a broad population of adolescents. The SEI classroom curriculum sought to engage students whether or not they had yet become sexually active. It is important to note, however, that the small percentage of students who were sexually experienced at baseline, fewer than one in five, limits the statistical power of tests of differential effectiveness.

Our analyses further aimed to take into account the effect of the classroom environment by including classroom level variables for gender and sexual experience. We found no contextual effect by either variable. That is, there were no additional changes to the outcome scores due to the proportion of students in each class who were female or sexually experienced.

### Implications for research and practice

This study offers a first look at the effectiveness of a rights-based sexuality education intervention for adolescents in U.S. schools and, as such, contributes to a greater understanding of this approach for those involved in sexuality education. Our findings are consistent with the small body of existing studies of interventions that have addressed similar concepts. For example, a quasi-experimental study of *The World Starts with Me*, a sexuality education program in secondary schools in Uganda emphasizing personal decision-making, social influences, gender equity, sexuality and rights, found a positive short-term impact on participating students’ beliefs about pregnancy prevention, norms about delaying sex, and self-efficacy about condom use relative to a comparison group of students [33]. A series of clinic-based sexual health interventions developed by DiClemente and colleagues have emphasized issues of gender, empowerment and healthy relationships, with evidence supporting their impact on both psychosocial mediators and behavioral outcomes for sexually active African American adolescent girls [30,46]. A similar intervention for Hispanic adolescent girls is under development [47]. The preliminary findings from the present SEI evaluation trial lend further support to the potential of rights-based approaches.

These findings also lend support to the existing conceptual definitions and theoretical frameworks guiding the development of rights-based sexuality education. The connections between individuals’ conceptions of gender and power in relationships and their sexual attitudes, behaviors, and health outcomes have been identified through prior research [28,48-50], and mechanisms for the influence of cultural and societal expectations about gender on decisions about relationships and sexual behaviors have been proposed [50-52]. In this study, we offer further support to these conclusions by showing that exposure to a rights-based curriculum can positively affect young people’s sexual knowledge, attitudes, and communication immediately following participation.

Finally, this study also brings attention to important issues of measurement and evaluation for sexuality education researchers. In 2012, the International Planned Parenthood Federation described the need to develop new measures of effectiveness for comprehensive sexuality education to balance broader outcomes, such as those related to gender and rights, with those related to public health [53]. Few such measures currently exist, especially for a school-based adolescent population in the U. S. For this trial, we developed and validated several new measures—such as attitudes about sexual relationship rights—that will enable future testing of hypothesized relationships between changes in knowledge, attitudes, and communication about gender, rights, and sexuality and changes in long-term sexual health outcomes.

### Strengths and limitations

This study has a number of strengths that support our interpretation of its results. These include the randomization of classroom- and school-level clusters to treatment conditions, the use of a standard control curriculum to mirror the typical provision of sexuality education to high school students, the validation of survey measures through pilot testing and psychometric analyses, sound fidelity of implementation, the strong follow-up rates without indication of differential attrition between the intervention and control groups, and the use of multilevel analytic models to account for the clustered design. In addition, the randomized evaluation trial was enhanced by rigorous formative research methods and the use of existing best practices to both develop the curriculum and assess the fidelity of its implementation [27,34].

These results should be considered in light of several limitations. First, the different lengths of the intervention and control curricula limit our ability to state conclusively that the SEI’s rights-based approach was responsible for the positive pattern of results. Given the study design, it not possible to disentangle the effects of curriculum length from content through statistical analyses. We believe that the SEI’s conceptual framework offers a strong explanation for the identified effects; nonetheless, it will be important for future research to examine this issue more closely. Second, as is common practice in school-based intervention studies, data were collected using self-report questionnaires which might be subject to response bias. Considerable efforts were made to support the validity and reliability of the survey measures, including the use of cognitive interviews during survey development to assess students’ comprehension and response processes [54], the inclusion of measures to examine students’ self-reported understanding, honesty, and carefulness in answering questions [55], and protocols to create a confidential environment during data collection. Third, for the three scales randomly administered to only half of the sample (sexual relationship rights with steady partner, sexual relationship rights with casual partner, and parent sexual communication), the effective sample size was half of what it was for the other scales, thereby reducing these scales’ statistical power to capture real effects. Yet, because these three scales all yielded statistically significant intervention effects, this concern was eliminated. Finally, these findings might not be generalizable to populations beyond the sample of urban, low-income, predominantly Hispanic high school students enrolled in public charter schools in the U.S. Although charter schools were employed in this evaluation, these were affiliated with the larger public school district and served comparable student populations. Future research is needed to determine whether the effects of the SEI are replicable in other contexts.

## Conclusions

This study provides evidence that the SEI improved students’ sex- and sexuality- related knowledge, attitudes, communication, and self-efficacy immediately following curriculum delivery. As a part of the first large-scale rigorous evaluation trial of a rights-based sexuality education intervention in the U.S., this study provides preliminary support to the theoretical foundations and potential for public health impact of this emerging intervention approach. Our results suggest that an intervention based on integrated theories of human rights, gender equality, and healthy sexual development can affect precursors to healthy sexual behavior among adolescents. Although much work is needed to understand the long-term effectiveness of the rights-based approach, the positive effects of the SEI on short-term psychosocial outcomes encourages further theoretical consideration, programmatic development, and empirical research to understand how this model could support and enhance adolescents’ healthy sexual development.

## Abbreviations

HIV: Human immunodeficiency virus
PPLA: Planned Parenthood Los Angeles
SEI: Sexuality Education Initiative
STI: Sexually transmitted infection
